# Pd-Modified LaFeO_3_ as a High-Efficiency Gas-Sensing Material for H_2_S Gas Detection

**DOI:** 10.3390/nano12142460

**Published:** 2022-07-18

**Authors:** Heng Zhang, Jing Xiao, Jun Chen, Yan Wang, Lian Zhang, Shuai Yue, Suyan Li, Tao Huang, Da Sun

**Affiliations:** 1 College of Physics and Electronic Engineering, Taishan University, Taian 271000, China; h_zhangabc@163.com (H.Z.); nankaichen@163.com (J.C.); joko365@163.com (Y.W.); alian_1987512@126.com (L.Z.); shuaiyue426@foxmail.com (S.Y.); lisuyantsu@163.com (S.L.); thuangdd@sina.com (T.H.); 2 Institute of Life Sciences & Biomedical Collaborative Innovation Center of Zhejiang Province, Wenzhou University, Wenzhou 325035, China; 3 Zhejiang Provincial Key Laboratory for Water Environment and Marine Biological Resources Protection, National & Local Joint Engineering Research Center for Ecological Treatment Technology of Urban Water Pollution, Wenzhou University, Wenzhou 325035, China

**Keywords:** Pd, LaFeO_3_, H_2_S, sensor, GC–MS

## Abstract

As a typical *p*-type semiconductor gas-sensing material, LaFeO_3_ has good response stability to H_2_S, but its responsiveness is low, and the detection limit is not low enough for large-scale use in the field of gas sensors. To obtain better performance, we synthesized Pd modified LaFeO_3_ using the sol–gel method. A total of 3 wt% of Pd–LaFeO_3_ with a high specific surface area had the highest response to H_2_S (36.29–1 ppm) at 120 °C, with relatively fast response–recovery times (19.62/15.22 s), and it had higher selectivity to H_2_S with other gases. Finally, we detected the H_2_S concentrations in the air around the shrimps, and the H_2_S concentrations that we obtained by the 3 wt% Pd–LaFeO_3_ in this study were within 10% of those obtained by GC–MS. According to the experimental results, noble-metal surface modification improves the performance of gas-sensing materials, and Pd–LaFeO_3_ has considerable potential in H_2_S detection.

## 1. Introduction

H_2_S is a colorless, highly toxic and acidic gas with a particular rotten egg smell, and even low concentrations of H_2_S can impair the human sense of smell. In high concentrations, it has no smell (because high concentrations paralyze the olfactory nerve). In addition, H_2_S is flammable and typically dangerous [1,2,3,4,5,6,7,8]. H_2_S gas is released during the breakdown of food, and it is also responsible for bad breath caused by periodontitis [8,9,10,11]. There are about 0.195 ppm H_2_S in the exhaled breath of a person with periodontitis and 0.105 ppm in that of a healthy person [12]. Using the nose as a means of detecting H_2_S can be fatal. Therefore, the timely detection of very low concentrations of H_2_S gas is important.

In recent years, the use of MOSs (metal oxide semiconductor) gas sensors to detect the concentrations of target gases has become increasingly popular. These are similar to smoke sensors in hotels, natural gas alarms in homes, etc. Some MOSs have excellent responses to gases, such as LaFeO_3_ [13,14,15,16], SmFeO_3_ [17,18,19,20,21], PrFeO_3_ [22,23], HoFeO_3_ [24], NdFeO_3_ [25], YCoO_3_ [26], BaSnO_3_ [27], ZnSnO_3_ [28] and YMnO_3_ [29]. For H_2_S, the gas-sensing materials are as follows: Pt–ZnO [30], Pd–ZnO [31], CuO/SnO_2_ [32], Pt–WO_3_ [33], WO_3_ [34,35,36], Pt–Fe_2_O_3_ [37], CuO/CuFe_2_O_4_ [38], Ag–SnO_2_ [39], YMnO_3_ [29], Sn–NiO [40], Au–SnO_2_ [41], Co_3_O_4_ [42], Pt–SnO_2_ [43], Ag–TiO_2_ [44],WO_3_ [45], Au–ZnO [46], ZnO/ZnSe [47], Pt–ZnO [48], CoFe_2_O_4_ [49], Pt–Zn_2_SnO_4_ [50], etc. MOSs, especially Pd–LaFeO_3_ perovskite materials, have the unique advantages of large specific surface areas and abundant active sites, which can promote the diffusion path and increase the adsorption of target gas molecules, thereby enhancing their sensing abilities. In particular, Pd–LaFeO_3_ is widely used in gas sensing, displaying excellent performance, a low detection limit, strong humidity adaptability and long-term stability. According to previously published studies, the response of Pd–LaFeO_3_ is as follows: 1.9–1 ppm acetone [51]; 1.2–100 ppm CO [52]. Although there are many reports on the detection of H_2_S by gas sensors, the performances of gas-sensing materials to H_2_S are generally not sufficient, and the detection limits cannot reach the PPB level, which is a challenge for realistic requirements.

The aim of this study was to obtain a gas-sensing material with a low detection limit and high response rate, selectivity and long-term stability. We synthesized Pd–LaFeO_3_ via the sol–gel method and sintered it at 800 °C. Pd–LaFeO_3_ had a high specific surface area and porosity, which are two important factors for gas-sensing materials to improve their responses to target gases. Compared with LaFeO_3_, Pd–LaFeO_3_ showed a higher response to the H_2_S gas and compared with other gases, 3 wt% Pd–LaFeO_3_ showed high selectivity for H_2_S. In addition, Pd nanoparticles as a catalyst greatly enhanced the surface activity of the gas-sensing materials and greatly shortened the response–recovery times. The innovation of this research is in the use of MOS gas sensors to explore the practical applications of H_2_S gas detection, which are rarely shown in other reports of MOS gas sensors. We detected the H_2_S concentrations in the air around shrimps using a gas sensor, and we compared them with the results obtained by the GC–MS method; the error was within 10%. According to the experimental results, Pd nanoparticles greatly improve the response of LaFeO_3_ to H_2_S gas, and the detection of H_2_S gas by a gas sensor is a feasible and effective method.

## 2. Materials and Methods

### 2.1. Preparation and Characterization of Nanocrystalline Pd–LaFeO_3_

**Figure 1:**
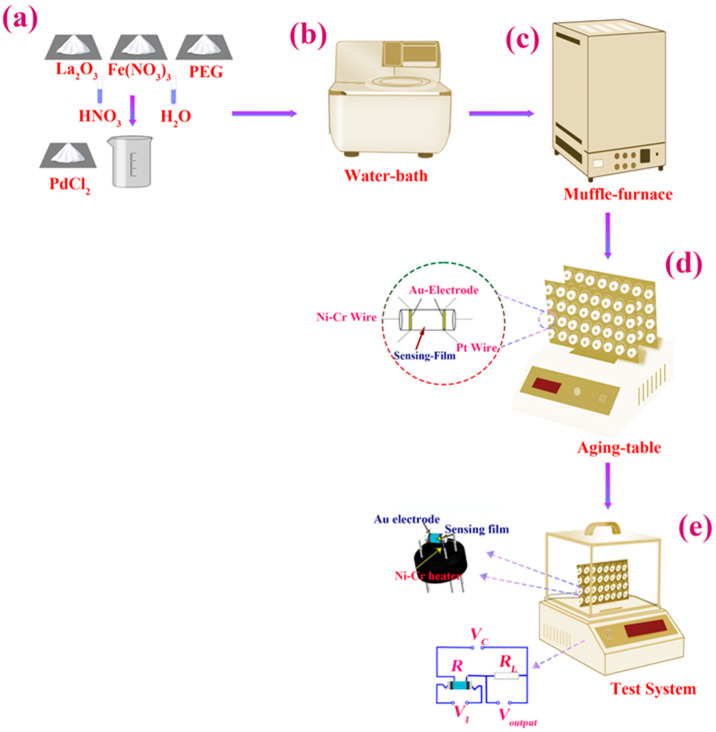
(**a**–**d**) Flowchart of Pd–LaFeO_3_ preparation; (**e**) gas-sensor-structure diagram and gas-sensor test system.

In Figure 1a, we present the chemical raw materials used in this work. First, we placed the weighed chemical materials (La_2_O_3_ (32.58 g), Fe(NO_3_)_3_ (48.4 g), PdCl_2_ (7.287 g), PEG (M_w_ 20,000, 60 g), HNO_3_ (97%, 500 mL) and deionized water (1000 mL)) in the beaker, and then we added the solution of HNO_3_ for the mixed dissolution over a period of 4 h (Figure 1b). The mixed solution sat for 2 h, and then we placed it in a water bath and stirred it at 80 °C for 24 h to obtain the mixed sol. Then, the mixed sol was removed and pre-sintered at 100 °C for 2 h in a muffle furnace. It was then gridded and sintered again at 800 °C for 6 h in the muffle furnace (Figure 1c). Finally, we obtained the Pd–LaFeO_3_ powder (approximately 38 g).

### 2.2. Fabrication and Measurement of Sensor

We mixed the Pd–LaFeO_3_ powder with deionized water to produce a paste, and then we placed the paste on a ceramic tube (Al_2_O_3_), approximately 2 mm in external diameter, 8 mm in length and 1.6 mm in internal diameter, with two electrodes installed at each end (Figure 1d). Then, we placed the whole prepared sensor on the aging table and aged it at 200 °C for 24 h (Figure 1d). After that, we tested the prepared sensor for the targeted gas in the gas-sensor test system (Figure 1e).

### 2.3. Ready-Made Sensor

In Figure 1e, we present the gas sensor structure diagram and the test circuit of the gas-sensor test system. The Ni–Cr wire was used to heat the sensing material to a higher operating temperature. Au electrodes and Pt wires were used to monitor the resistance of gas-sensing materials in real-time. $V_{C}$ is the supply voltage, which was kept constant at 5 V; $V_{1}$ is the voltage at both ends of the gas sensor; $R_{l}\;$ is the value of the variable resistor; $V_{output}$ is the voltage across $R_{l}$; R is the resistance value of the sensor. We calculated R using the following formula:

(1) R= v1voutputRl.

The gas-sensing response (*S*) was defined as $R_{g}/R_{a}$: $R_{g}$ is the resistance of the sensor in the tested gas, and $R_{a}$ is the resistance of the sensor when it is in the air. We defined the response time as the time taken to attain 90% of the maximum value in the ascending phase and the recovery time as the time taken to regain 10% of the base value in the descending phase. The experimental environment was as follows: RH, 20%; environment temperature, 20 °C.

## 3. Results

### 3.1. Material Characterization

**Figure 2:**
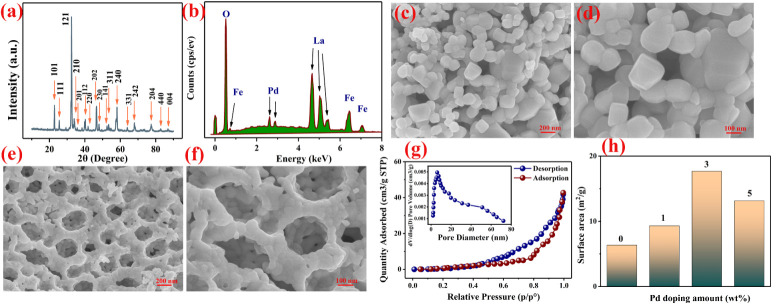
(**a**) XRD pattern of 3 wt% Pd–LaFeO_3_; (**b**) EDS pattern of 3 wt% Pd–LaFeO_3_; (**c**,**d**) SEM spectra of pure LaFeO_3_; (**e**,**f**) SEM spectra of 3 wt% Pd–LaFeO_3_; (**g**) N_2_ adsorption–desorption isotherms and pore size distributions (the inset) for 3 wt% Pd–LaFeO_3_ nanocomposite; (**h**) specific surface areas of LaFeO_3_ with different amounts of Pd.

In Figure 2a, we present the X-ray diffraction analysis (XRD) (Bruker D8 ADVANCE with a CuKα amount of 1.5405 Å at 40 kV and 40 mA, Berlin, Germany) of the 3 wt% Pd–LaFeO_3_. Compared with the standard card (PDF card: 37–1493), the Pd–LaFeO_3_ shows a single phase. We can calculate the average particle size by the Scherrer method. The Scherrer equation is as follows:

(2) $$D=\frac{k\lambda }{\beta \cos\theta }$$

where λ is the wavelength of the X-ray, β is the integral width of the diffraction peaks and θ is the Bragg diffraction angle. The average particle size of Pd–LaFeO_3_ is about 68.7 nm. Because of the low amount of Pd, the characteristic peak was not reflected in the XRD pattern, so we performed EDS (energy dispersive spectroscopy) for 3 wt% Pd–LaFeO_3_ to confirm the presence of Pd. As can be seen in Figure 2b, Pd was present in the material. The atomic compositions of the elements in all samples are shown in Table 1. No other impure elements were present in any of the samples. Figure 2c–f shows the scanning electron microscopy (SEM) (HITACHI SU8010 8.0 kV, Tokyo, Japan) spectra of LaFeO_3_ and 3 wt% Pd–LaFeO_3_ under different magnifications. The unmodified LaFeO_3_ has a common perovskite structure, and the 3 wt% Pd–LaFeO_3_ has a network structure.

To understand which microstructure was more favorable to the properties of the gas-sensing material, we needed to ascertain which structure had a higher specific surface area and porosity. We further analyzed the specific surface area and porosity of the 3 wt% Pd–LaFeO_3_ hollow nanofibers by nitrogen adsorption–desorption measurement. In Figure 2g, we present the BET curves for 3 wt% Pd–LaFeO_3_ and the corresponding Barrett–Joyner–Halenda (BJH) pore size distribution (inset). The specific surface area of 3 wt% Pd–LaFeO_3_ is 17.53 m^2^/g, and the average pore size is 13.6 nm. We present the specific surface areas of LaFeO_3_ with different amounts of Pd in Figure 2h. When the amount of Pd was 3 wt%, the composite powder obtained the largest specific surface area. Because Pd nanoparticles can inhibit the growth of the MOS grain, the smaller the grain size, the larger the specific surface area. However, when the amount of Pd is too high, the particles appear in a small range of agglomerations, and the specific surface area of the material decreases. The specific surface area is an important factor for sensing the properties of materials. A large specific surface area can provide more adsorption sites, which enhances the reactions between the sensing material and gas molecules, which results in a high response to the test gas.

**Figure 3:**
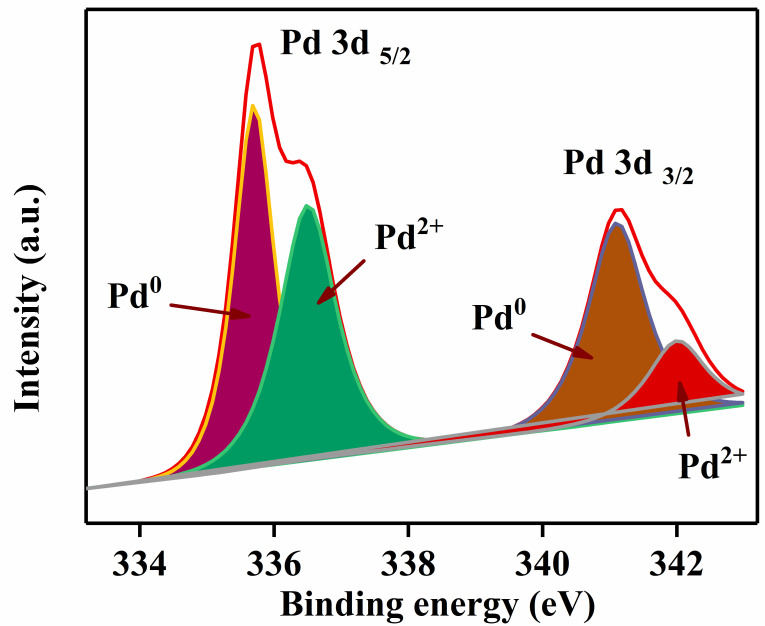
X-ray photoelectron spectroscopy (XPS) of Pd in the material.

The X-ray photoelectron spectroscopy (XPS) spectra for the presence of Pd in the material are shown in Figure 3. The spectra consist of two peaks, Pd 3d_5/2_ (335.8 eV) and Pd 3d_3/2_ (341.3 eV). The Pd 3d_5/2_ peak consists of a high-intensity peak at 335.6 eV, related to Pd^0^, and a low-intensity peak at 336.5 eV, related to Pd^2+^. The Pd 3d_3/2_ also consists of two peaks, at 341.1 and 341.9 eV. Additionally, this can also indicate Pd is not doped into the lattice of LaFeO_3_; therefore, Pd–LaFeO_3_ is a composite material.

### 3.2. Gas-Sensing Performance

**Figure 4:**
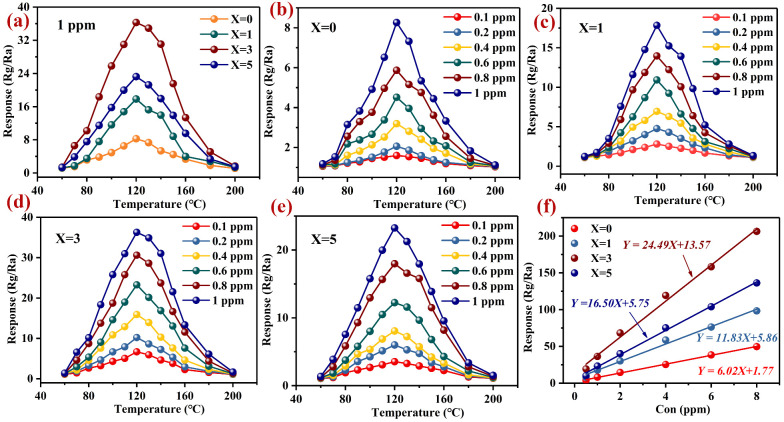
(**a**) Responses of Pd–LaFeO_3_ to 1 ppm H_2_S with operating temperatures; (**b**–**e**) responses of Pd–LaFeO_3_ to different concentrations of H_2_S with operating temperature; (**f**) linear relationship between the responses and H_2_S concentrations; X refers to the amount of Pd.

In Figure 4a, we present the response curves of the LaFeO_3_ with different amounts of Pd to 1 ppm H_2_S with the operating temperatures. For all the samples, we obtained the highest responses at 120 °C. The highest responses to 1 ppm H_2_S were 8.26 (0 wt% Pd), 17.85 (1 wt% Pd), 36.29 (3 wt% Pd) and 23.26 (5 wt% Pd). The responses were more than four times higher than before modification with Pd. In Figure 4b–e, we present the responses of all the samples to 0.1–1 ppm H_2_S with the operating temperatures. For any H_2_S concentration, the optimum operating temperature was 120 °C. In Table 2, we present the responses of the LaFeO_3_ with different amounts of Pd.

The relationship between the material’s sensitivity and the gas concentration is important, and we can use a high fitting relationship to predict the response value at a given gas concentration. In Figure 4f, we can see the relationship between the responses of Pd–LaFeO_3_ to multiple H_2_S concentrations. For both the unmodified and modified Pd–LaFeO_3_, the responses have a good linear relationship with the gas concentrations and all the $R^{2}$ values are greater than 98%.

**Figure 5:**
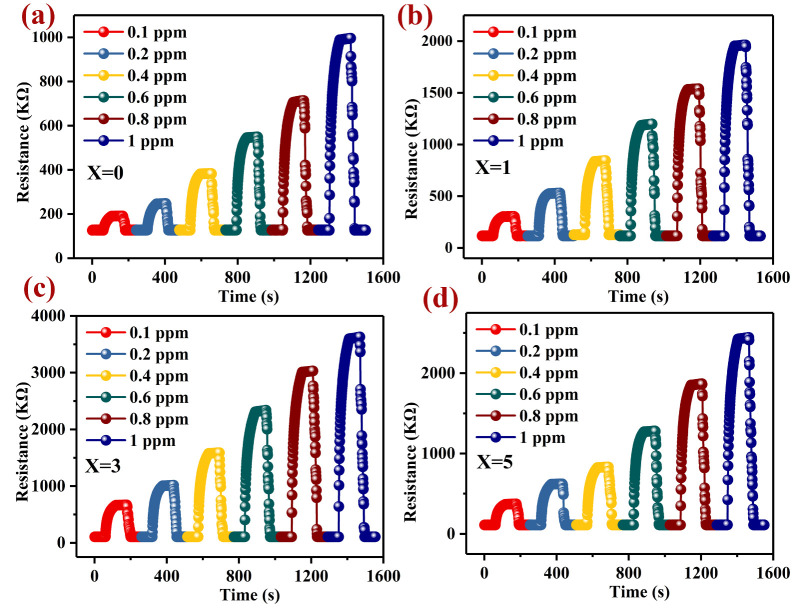
The dynamic resistances of LaFeO_3_ with different amounts of Pd to different concentrations (0.1–1 ppm) of H_2_S; X refers to the amount of Pd (**a**–**d**).

**Figure 6:**
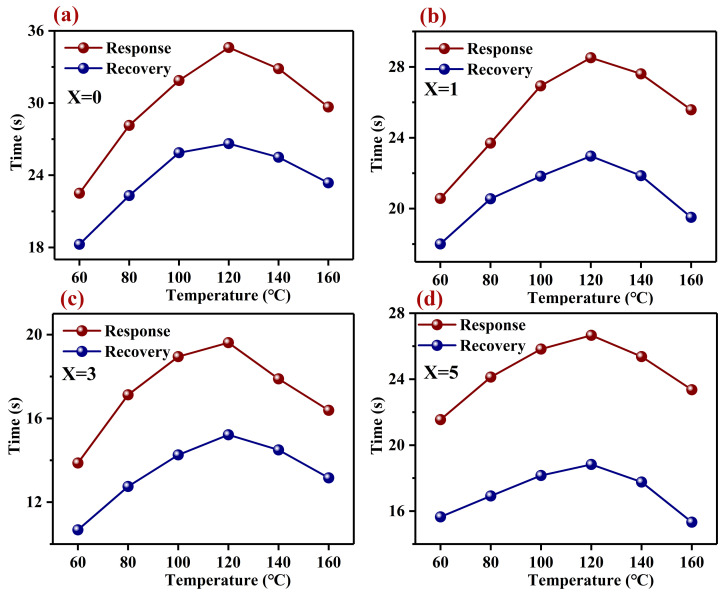
The response and recovery times of Pd–LaFeO_3_ to 1 ppm H_2_S at different operating temperatures; X refers to the amount of Pd (**a**–**d**).

Repeatability is another important property that determines whether a gas sensing material is sufficient or not. In Figure 5a–d, we present the repeatability of the responses to different concentrations (0.1–1 ppm) of H_2_S gas for Pd–LaFeO_3_. All the repeated processes went as follows: When the resistance value of the gas-sensing material was stabilized, we injected the H_2_S gas into the reaction chamber, and the resistance of the material increased immediately. After a period of time, the resistance stabilized, we removed the H_2_S gas, the resistance of the material decreased immediately and we could restore it to the initial state. For the H_2_S gas with different concentrations, we could restore the resistance of the gas-sensing material to the initial value after every time the H_2_S gas was removed, which indicated that the material had excellent repeatability. The response–recovery times of all the samples were different at different operating temperatures, which indicated that the operating temperature affected the chemical reaction on the material’s surface. We present the response–recovery times of all the samples in Table 3 and Figure 6a–d. The response–recovery times increased with the operating temperature before it reached 120 °C, and after 120 °C, the response–recovery times decreased with the further increases in the operating temperature. Before the optimum operating temperature, the adsorption rate of the gas molecules was higher than the desorption rate, and the number of adsorbed oxygen ions and H_2_S gas molecules on the material’s surface increased, which led to the increase in the reaction time. With the increase in the operating temperature, the adsorption and desorption rates maintained a balance at the optimum operating temperature, and the number of H_2_S gas molecules and adsorbed oxygen ions on the material’s surface reached the maximum. At this operating temperature, the reaction time also reached the maximum. With the further increase in the operating temperature, the desorption rate of the gas molecules was higher than the adsorption rate, the reaction reactants became fewer and the reaction time became shorter. In addition, the response–recovery times of the 3 wt% Pd–LaFeO_3_ were reduced by two times that of the pure LaFeO_3_.

**Figure 7:**
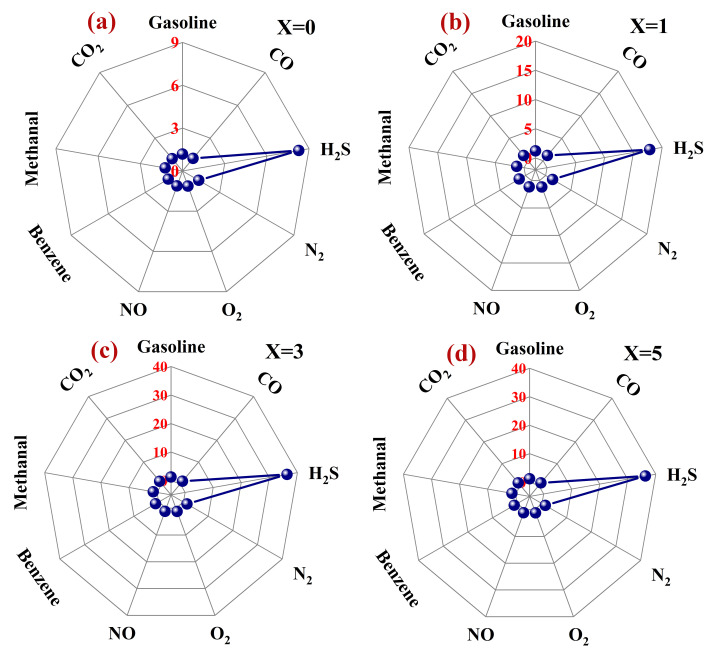
Selectivity comparison of 0–5 wt% Pd–LaFeO_3_ to 1 ppm H_2_S and several other common gases in a person’s exhaled breath; X refers to the amount of Pd (**a**–**d**).

In practical application, certain gases are commonly detected in mixtures, especially H_2_S gas in a real person’s exhaled breath. Therefore, the selectivity of a gas-sensing material to a certain gas determines its practical application value. We present a selectivity comparison between 0–5 wt% Pd–LaFeO_3_ to 1 ppm H_2_S and several other common gases in a person’s exhaled breath in Figure 7a,d. Compared with other gases, Pd–LaFeO_3_ has a high selectivity for H_2_S gas. For N_2_, O_2_, NO, CO_2_, CO and other common gases in a person’s exhaled breath, the responses can be negligible, and the H_2_S in the exhaled breath can more accurately be detected.

**Figure 8:**
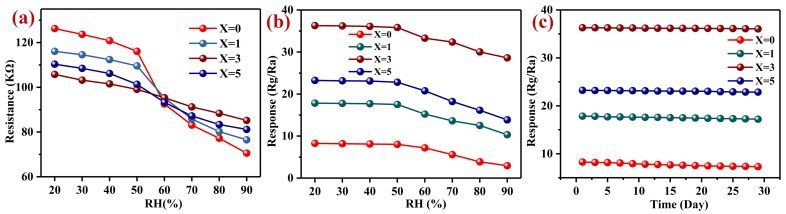
(**a**) Resistance curves of Pd–LaFeO_3_ with RH; (**b**) response curves of Pd–LaFeO_3_ with RH; (**c**) response curves of Pd–LaFeO_3_ over time; X refers to the amount of Pd.

The relative humidity (RH) in the environment is also a factor that cannot be ignored in the application of gas sensors. In Figure 8a, we show the resistance changes of Pd–LaFeO_3_ with the RH at 120 °C. For Pd–LaFeO_3_, the resistance decreased with the RH, but the proportions of these decreases were different. In the 20–90% RH range, the proportions of the decreases were: 44.13% (0 wt% Pd), 34.1% (1 wt% Pd), 19.46% (3 wt% Pd) and 26.46% (5 wt% Pd), which means that the resistance of 3 wt% Pd–LaFeO3 had the greatest RH adaptability. The RH can also affect the response of the gas-sensing material to the target gas. It has been demonstrated that oxygen (O_2_) can capture the electrons from the surface of the materials to generate adsorbed oxygen (O_2_^−^) which can further react with the surface-adsorbed water molecules (H_2_O) to produce hydroxyl groups (OH^–^). Their reactions can be expressed as follows [53,54,55,56,57]:

(3) $$e^{–}+{\;O}_{2}\;\to {\;O}_{2}^{–};$$

(4) $$O_{2}^{–}+{\;H}_{2}O\;\to {\;\mathrm{HO}}_{2}^{∙}+{\mathrm{OH}}^{–}.$$

Based on the hydrogen bonding effect [53,57], the greater the number of hydroxyl groups, the more surface-absorbed water molecules and adsorbed oxygen there are, and the more holes are generated accordingly. Therefore, the resistance decreased with the RH.

In Figure 8b, we present the responses of Pd–LaFeO_3_ to 1 ppm H_2_S with RH. The responses decreased with the RH. Moreover, before 50% RH, the RH did not have a significant effect on the responses. However, after 50% RH, the responses decreased sharply, which means that the gas sensor used in this study can be used in low-RH environments without considering the influence of the RH. This feature greatly expands its practical applications in the field.

Long-term stability is another important property of gas-sensing materials. The higher the long-term stability, the longer the replacement cycle of the gas-sensing material, and the more economical and energy advantages it has. In Figure 8c, we show the long-term stability of Pd–LaFeO_3_ over 30 days (pH = 6.8–7.3). The experimental data were recorded every two days. All the responses decreased slightly with time, but the proportions of the decreases were different, as follows: 11.45% (0 wt% Pd), 3.6% (1 wt% Pd), 0.65% (3 wt% Pd) and 1.63% (5 wt% Pd). The long-term stability of the 3 wt% Pd–LaFeO_3_ was more than 17 times that of the pure LaFeO_3_. LaFeO_3_ with Pd presented greater advantages in terms of long-term stability.

## 4. Sensing-Mechanism Analysis

**Figure 9:**
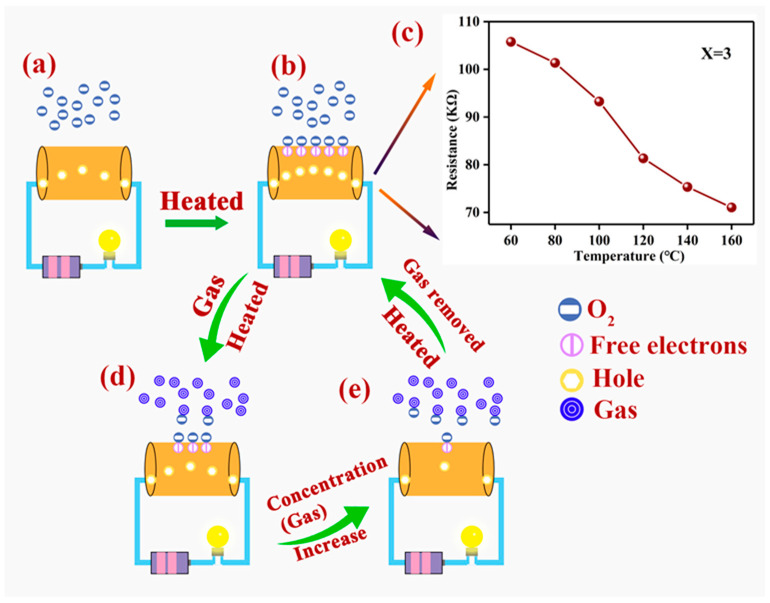
Reaction mechanism of the whole experiment in this work: (**a**) charge state on the surface of the material at room temperature; (**b**) charge state on the surface of the material under high temperature; (**c**) resistance curve of 3 wt% Pd–LaFeO_3_ with operating temperatures; (**d**) change in charge state on surface of material after gas injection under high temperature; (**e**) change in charge state on surface of material with gas concentration under high temperature.

In Figure 9, we show the reaction mechanism of the whole experiment in this work. For a *p*-type semiconductor, the main carrier of Pd–LaFeO_3_ is the hole ($h^{•}$) (Figure 9a). The oxygen molecules that were adsorbed onto the surface of the Pd–LaFeO_3_ continuously captured the electrons from the material, which caused an increase in the number of holes (Figure 9b). The rate at which the oxygen molecules captured electrons was slow at room temperature and had little effect on the resistance value. However, as the operating temperature gradually increased, the capture rating on the surface of Pd–LaFeO_3_ increased. Therefore, the resistance decreased with the operating temperature (Figure 9c). In addition, the word function of the Pd was greater than the LaFeO_3_, the free electron on the surface of the LaFeO_3_ was much easier to transfer to the Pd nanoparticles and forming a depletion layer increased the material’s resistance when it was in the air.

The reaction between the oxygen molecules and free electrons on the surface of the Pd–LaFeO_3_ is as follows [58,59,60]:

(5) $$O_{2}+{\;e}^{-}\;\to {\;O}_{2}^{-}\left(\mathrm{ads}\right)+{\;h}^{+};$$

(6) $$O_{2}^{-}\left(\mathrm{ads}\right)+{\;e}^{-}\;\to \;2O^{-}\left(\mathrm{ads}\right)+{\;h}^{+}.$$

The ads refer to the state of the adsorbed oxygen on the surface of the LaFeO_3_.

When the H_2_S gas molecule was introduced, it was adsorbed onto the surface of the LaFeO_3_ and reacted with the oxygen ions (Figure 9d). The adsorption and desorption on the surface of the Pd–LaFeO_3_-to-H_2_S gas molecules occurred simultaneously. The rates of adsorption and desorption increased with the operating temperature, and the rate of adsorption was greater than the rate of desorption before the operating temperature reached the optimum temperature. Therefore, the number of adsorbed H_2_S molecules on the surface of the material increased, and the reaction between the H_2_S molecules and oxygen ions was more intense, which resulted in response to the increase. When the operating temperature exceeded the optimum temperature, the rate of the adsorption of the Pd–LaFeO_3_-to-H_2_S molecules was lower than the rate of desorption, and the intensity of the reaction between the H_2_S molecules and oxygen ions was reduced, which caused the response to decrease. Moreover, at the optimum temperature, as the concentration of H_2_S gas molecules increased, this increased the number of adsorbed H_2_S molecules on the surface of the Pd–LaFeO_3_ and caused the response to increase (Figure 9e). However, the number of free electrons on the surface of the Pd–LaFeO_3_ is not infinite, and the energy required to make an electronic transition within Pd–LaFeO_3_ also increases. Therefore, the response ($R_{g}/R_{a}$) increased with the concentration of H_2_S gas molecules, but the rate of increase declined. In addition, when the free electron was released from the adsorbed oxygen ions to the Pd–LaFeO_3_, the width of the LaFeO_3_ in the depletion layer narrowed, which was caused by Pd, which resulted in a greater resistance change.

The reaction between the H_2_S molecules and oxygen ions is as follows [30,31]:

(7) $$2H_{2}S+3O_{2}^{-}\;\left(\mathrm{ad}\right)\;\to 2{\mathrm{SO}}_{2}+2H_{2}O+3e^{-}$$

(8) $$e^{-}+h^{+}\;\to \mathrm{null}$$

## 5. Application in the Detection of H_2_S

**Figure 10:**
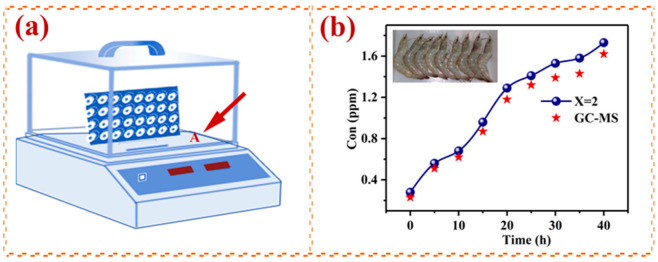
(**a**) Experimental setup used for H_2_S detection of the emission of shrimps; (**b**) dynamic curve of H_2_S concentration around shrimp with time.

The accurate and quick assessment of the decomposition of meat and seafood is important. H_2_S is among the most important gases released in the decomposition of food [30]. In this study, we detected the H_2_S concentrations around shrimp over time using a gas sensor (Figure 10a) and GC–MS (GCMS-QP2020 NX), and we present the results in Figure 10b. There were nine shrimps in the experimental apparatus, each about 10–16 cm in length. We placed them on a plate in a closed test system (about 28 L). Point A refers to where the shrimp were placed. The concentration of H_2_S increased with the time from death, and the concentration of H_2_S measured by the gas sensor was greater than that measured by GC–MS at any time, which indicates that there were other gases in the air surrounding the shrimp that could have affected the gas sensor, but the effect was very small. We compared the H_2_S concentrations measured by the two methods, and the error was within 10%. We present the results in Table 4.

## 6. Conclusions

In this study, we synthesized Pd modified LaFeO_3_ with a large specific surface area and high porosity by the sol–gel method, which improved the response to a certain extent. According to the experimental results, the optimum Pd content is 3 wt%. The response of the 3 wt% Pd–LaFeO_3_ to H_2_S was more than four times higher than before modification with Pd, and the long-term stability was more than 17 times that of the pure LaFeO_3_. Moreover, the response–recovery times of the 3 wt% Pd–LaFeO_3_ were reduced by two times those of the pure LaFeO_3_. In addition, the use of Pd modification as a catalyst greatly improved the RH adaptability and selectivity of the material. Finally, the Pd–LaFeO_3_ was accurate in detecting the concentration of H_2_S gas in the air around the shrimp, with an error of less than 10%, compared with the results obtained by GC–MS. According to the experimental results, noble metal surface modification improves the performance of gas-sensing materials, and Pd–LaFeO_3_ has great potential for H_2_S detection.

## Figures and Tables

**Table 1:** The atomic compositions (%) of the elements in all samples.

	Pd	La	Fe	O
0	0	19.73	23.68	56.59
1	0.45	19.85	19.99	59.71
3	1.40	19.78	19.70	59.12
5	2.35	19.46	19.56	58.63

**Table 2:** Response of LaFeO_3_ with different amounts of Pd to H_2_S gas.

	Con (ppm)	0.1	0.2	0.4	0.6	0.8	1
Pd (wt%)							
0		1.59	2.06	3.19	4.52	5.87	8.26
1		2.8	4.76	6.94	10.93	13.97	17.85
3		6.69	10.22	15.93	23.27	30.62	36.29
5		3.53	5.98	8.06	12.25	17.98	23.25

**Table 3:** The response and recovery times of Pd–LaFeO_3_ to 1 ppm H_2_S at different operating temperatures.

	T (°C)	60	80	100	120	140	160	Pd (wt%)
Time (s)								
Response		22.5	28.15	31.88	34.61	32.86	29.68	0
Recovery		18.26	22.31	25.88	26.63	25.5	23.37	0
Response		20.58	23.7	26.93	28.52	27.61	25.58	1
Recovery		18.01	20.56	21.83	22.97	21.86	19.51	1
Response		13.87	17.13	18.95	19.62	17.89	16.39	3
Recovery		10.68	12.75	14.26	15.22	14.5	13.16	3
Response		21.55	24.13	25.83	26.66	25.37	23.36	5
Recovery		15.65	16.92	18.16	18.83	17.76	15.33	5

**Table 4:** Concentration of H_2_S obtained by gas sensor and GC–MS.

	Time (h)	0	5	10	15	20	25	30	35	40
Method										
Gas sensor		0.28	0.56	0.68	0.96	1.29	1.41	1.53	1.58	1.73
GC–MS		0.23	0.51	0.62	0.87	1.18	1.32	1.39	1.43	1.62

## Data Availability

Not applicable.
